# Supplementary material for: Does Inter-Organellar Proteostasis Impact Yeast Quality and Performance During Beer Fermentation?
Source: Front Genet. 2020 Jan 31;11:2. doi: 10.3389/fgene.2020.00002 (PMC7006503; doi:10.3389/fgene.2020.00002)
Supplement: Supplementary file 1 [file DataSheet_1.pdf]

Experimental procedures

DNA microarray gene expression and gene ontology analysis

DNA microarray gene expression (GSE) datasets (GSE9423, GSE10205, and GSE16376) comparing lager yeast CB11 strain (*Saccharomyces pastorianus*) during fermentation and propagation in different times (Table S1 and Figure S1) (Gibson et al., 2008) were obtained from Gene Expression Omnibus (GEO) database [http://www.ncbi.nlm.nih.gov/gds] (Table S1). In this study, the definitions of fermentation and propagation are the same used in the work by Gibson et al. (2008), where 'fermentation' is the process when beer is made from wort by yeast fermentation in absence of oxygen, while 'propagation' is defined as the process in which a sufficient yeast biomass is generated for beer production using wort as liquid media constantly supplied with molecular oxygen.

Table S1. Gene expression datasets (GSEs) used in this work.

Source[a]	GEO samples files	Sample name	Sample	Organism	Strain
GSE9423	GSM239499	Fermentation 30 hours	Ferm_30h_B	*Saccharomyces pastorianus*	CB11
GSE9423	GSM239503	Fermentation 60 hours	Ferm_60h_C	*Saccharomyces pastorianus*	CB11
GSE9423	GSM239512	Propagation 30 hours	Prop_30h_C	*Saccharomyces pastorianus*	CB11
GSE9423	GSM239504	Fermentation 8 hours	Ferm_8h_A	*Saccharomyces pastorianus*	CB11
GSE9423	GSM239514	Propagation 8 hours	Prop_8h_B	*Saccharomyces pastorianus*	CB11
GSE9423	GSM239501	Fermentation 60 hours	Ferm_60h_A	*Saccharomyces pastorianus*	CB11
GSE9423	GSM239510	Propagation 30 hours	Prop_30h_A	*Saccharomyces pastorianus*	CB11
GSE9423	GSM239515	Propagation 8 hours	Prop_8h_C	*Saccharomyces pastorianus*	CB11
GSE9423	GSM239506	Fermentation 8 hours	Ferm_8h_C	*Saccharomyces pastorianus*	CB11
GSE9423	GSM239505	Fermentation 8 hours	Ferm_8h_B	*Saccharomyces pastorianus*	CB11
GSE9423	GSM239511	Propagation 30 hours	Prop_30h_B	*Saccharomyces pastorianus*	CB11
GSE9423	GSM239502	Fermentation 60 hours	Ferm_60h_B	*Saccharomyces pastorianus*	CB11
GSE9423	GSM239508	Propagation 0 hours	Prop_0h_B	*Saccharomyces pastorianus*	CB11
GSE9423	GSM239500	Fermentation 30 hours	Ferm_30h_C	*Saccharomyces pastorianus*	CB11
GSE9423	GSM239507	Propagation 0 hours	Prop_0h_A	*Saccharomyces pastorianus*	CB11

Source[a]	GEO samples files	Sample name	Sample	Organism	Strain
GSE9423	GSM239509	Propagation 0 hours	Prop_0h_C	*Saccharomyces pastorianus*	CB11
GSE9423	GSM239513	Propagation 8 hours	Prop_8h_A	*Saccharomyces pastorianus*	CB11
GSE10205	GSM257787	Fermentation 102 hours	Ferm_102h_A	*Saccharomyces pastorianus*	CB11
GSE10205	GSM257776	Fermentation 8 hours	Ferm_8h_A	*Saccharomyces pastorianus*	CB11
GSE10205	GSM257778	Fermentation 8 hours	Ferm_8h_C	*Saccharomyces pastorianus*	CB11
GSE10205	GSM257789	Fermentation 102 hours	Ferm_102h_C	*Saccharomyces pastorianus*	CB11
GSE10205	GSM257780	Fermentation 30 hours	Ferm_30h_B	*Saccharomyces pastorianus*	CB11
GSE10205	GSM257782	Fermentation 60 hours	Ferm_60 h_B	*Saccharomyces pastorianus*	CB11
GSE10205	GSM257786	Fermentation 80 hours	Ferm_80h_C	*Saccharomyces pastorianus*	CB11
GSE10205	GSM257785	Fermentation 80 hours	Ferm_80h_B	*Saccharomyces pastorianus*	CB11
GSE10205	GSM257781	Fermentation 60 hours	Ferm_60h_A	*Saccharomyces pastorianus*	CB11
GSE10205	GSM257783	Fermentation 60 hours	Ferm_60h_C	*Saccharomyces pastorianus*	CB11
GSE10205	GSM257784	Fermentation 80 hours	Ferm_80h_A	*Saccharomyces pastorianus*	CB11
GSE10205	GSM257777	Fermentation 8 hours	Ferm_8h_B	*Saccharomyces pastorianus*	CB11
GSE10205	GSM257779	Fermentation 30 hours	Ferm_30h_A	*Saccharomyces pastorianus*	CB11
GSE10205	GSM257788	Fermentation 102 hours	Ferm_102h_B	*Saccharomyces pastorianus*	CB11
GSE16376	GSM410831	Propagation 0 hours	Prop_0h_A	*Saccharomyces pastorianus*	CB11
GSE16376	GSM410832	Propagation 0 hours	Prop_0h_B	*Saccharomyces pastorianus*	CB11
GSE16376	GSM410833	Propagation 0 hours	Prop_0h_C	*Saccharomyces pastorianus*	CB11
GSE16376	GSM410834	Propagation 4 hours	Prop_4h_A	*Saccharomyces pastorianus*	CB11
GSE16376	GSM410835	Propagation 4 hours	Prop_4h_B	*Saccharomyces pastorianus*	CB11
GSE16376	GSM410836	Propagation 4 hours	Prop_4h_C	*Saccharomyces pastorianus*	CB11
GSE16376	GSM410837	Propagation 8 hours	Prop_8h_A	*Saccharomyces pastorianus*	CB11
GSE16376	GSM410838	Propagation 8 hours	Prop_8h_B	*Saccharomyces pastorianus*	CB11
GSE16376	GSM410839	Propagation 8 hours	Prop_8h_C	*Saccharomyces pastorianus*	CB11
GSE16376	GSM410840	Propagation 30 hours	Prop_30h_A	*Saccharomyces pastorianus*	CB11
GSE16376	GSM410841	Propagation 30 hours	Prop_30h_B	*Saccharomyces pastorianus*	CB11
GSE16376	GSM410842	Propagation 30 hours	Prop_30h_C	*Saccharomyces pastorianus*	CB11

[a]In blue color, the GSE9423 used for single DNA microarray analysis. In red, the GSE10205 and GSE16376 used for DNA microarray meta-analysis.

All statistical analyses on transcriptome data were performed using the R platform [https://www.r-project.org] and the following packages: (i) GEOquery for data matrix importing and parsing (Davis and Meltzer, 2007); (ii) arrayQualityMetrics for microarray quality analysis (Kauffmann et al., 2009) and (iii) limma for differentially expressed gene (DEG) analysis (Ritchie et al., 2015) (Figure S1). The significance of DEGs was determined by False Discovery Rate (FDR) algorithm, implemented in limma package (Ritchie et al., 2015). Beer fermentation-associated DEGs from DNA microarray single- (GSE9423) and meta-analysis (GSE10205 versus GSE16376) with mean $|\text{logFC}| \geq 2.0$, logFC standard deviation (SD) < 1.0 and FDR < 0.05 were selected for gene ontology analyses and protein subcellular localization (Figure S1). To perform the gene ontology analysis, DEGs were specifically filtered for annotated proteostasis- and chaperones-linked genes using data from *Saccharomyces* Genome Database (Figure S1). Further, the proteostasis- and chaperones-linked DEGs obtained from DNA microarray single- and meta-analysis were applied to select a list of commonly observed DEGs in both analysis, and were called as proteostasis Pan-DEGs (Figure S1). The major biological processes and cellular component associated to proteostasis- and chaperones-linked DEGs lists from DNA microarray single- and meta-analysis and Pan-DEGs were further determined using the R package clusterProfile and *Saccharomyces cerevisiae* protein data from UniProt (Yu et al., 2012) (Figure S1). The degree of functional enrichment for a given biological process category was quantitatively assessed (p-value < 0.01) using a hypergeometric distribution. Multiple test correction was also assessed by applying FDR algorithm (Benjamini and Hochberg, 1995) at a significance level of $p < 0.05$. Semantic comparison among biological processes and cellular component associated to DEGs were made using R package GOSemSim (Yu et al., 2010) using false discovery rate (FDR) < 0.01 and q-value < 0.05 (Figure S1). Networks containing the subcellular targets of proteostasis- and chaperones-associated DEGs from DNA microarray single analysis, meta-analysis and Pan-DEGs were generated using the R package igraph and Cytoscape 3.7.2 (Shannon et al., 2003; Csardi and

Nepusz, 2006) (Figure S1). Heatmaps combining proteostasis- and chaperones-associated DEGs from DNA microarray single- and meta-analysis and Pan-DEGs values and GOs were designed with R package ComplexHeatmap (Gu et al., 2016), where rows and columns were grouped using Euclidean distance method and complete linkage (Figure S1). All Figures displayed in this supplementary material as well as in the manuscript can be downloaded at https://github.com/bonattod/Proteostasis_data_analysis.git

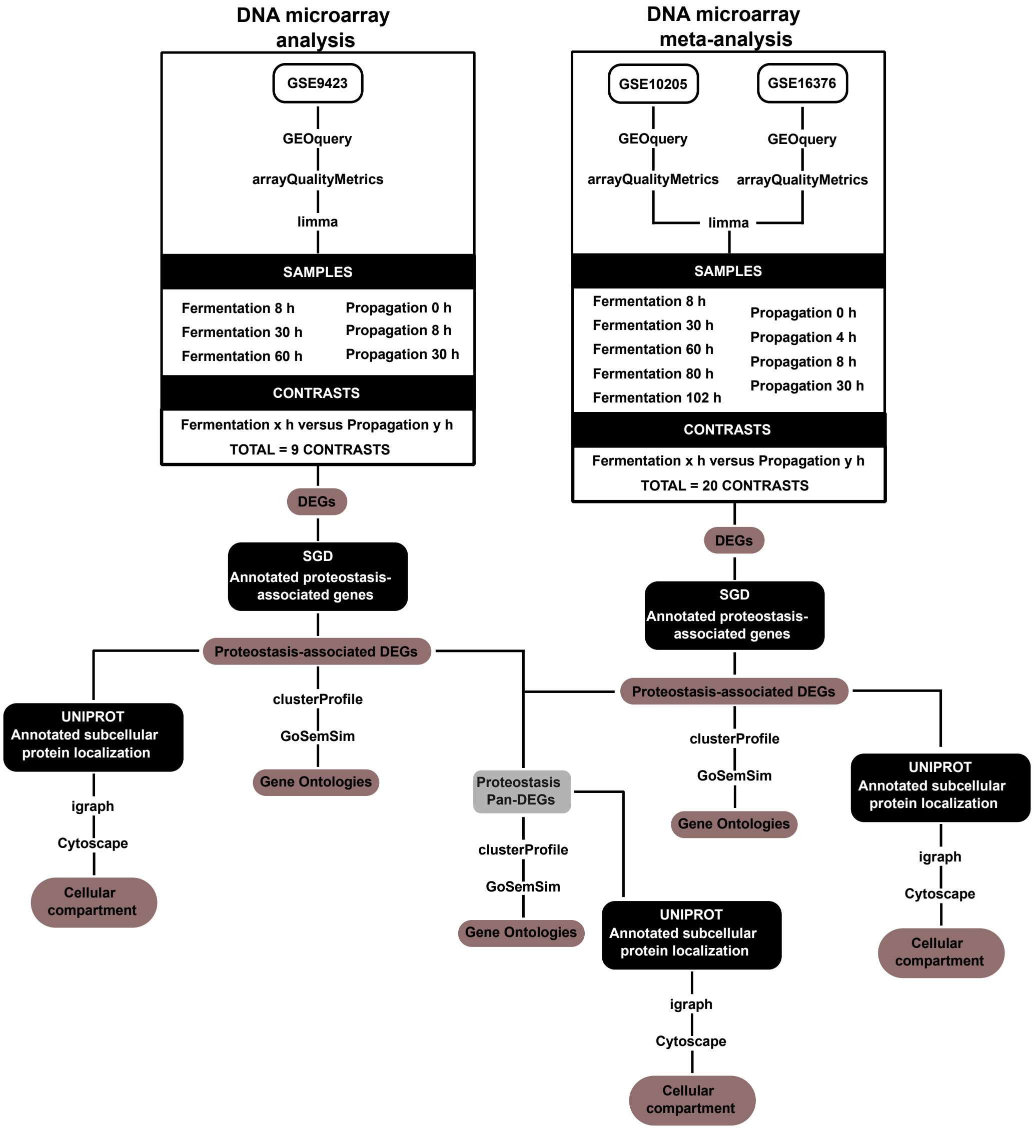

Figure S1. Experimental design used in DNA microarray single and meta-analysis. Abbreviation: differential by expressed genes (DEGs); *Saccharomyces* Genome Database (SGD); Universal Protein Resource (UniProt).

Supplementary results

DNA microarray single- and meta-analysis

Data gathered from DNA microarray single analysis (GSE9423) showed a low number of overexpressed and underexpressed DEGs comparing the very beginning of propagation condition (0 hour) with different times of fermentation (8, 30, and 60 hours) (Figure S2A), while the number of DEGs in fermentation compared to different propagation times (8 and 30 hours) dramatically increased (Figure S2A). A similar result was also observed for DNA microarray meta-analysis comparing different times of fermentation (GSE10205) and propagation (GSE16376) (Figure S2B), where the number of DEGs was low when yeast cells in the first hours of propagation (0 and 4 hours) were compared with yeasts in different times of fermentation (from 8 to 102 hours). Additionally, the number of DEGs in both DNA microarray single- and meta-analysis sharply increased when cells in different times of fermentation were compared with the same yeast strain after 8 hours of propagation (Figures S2A and B).

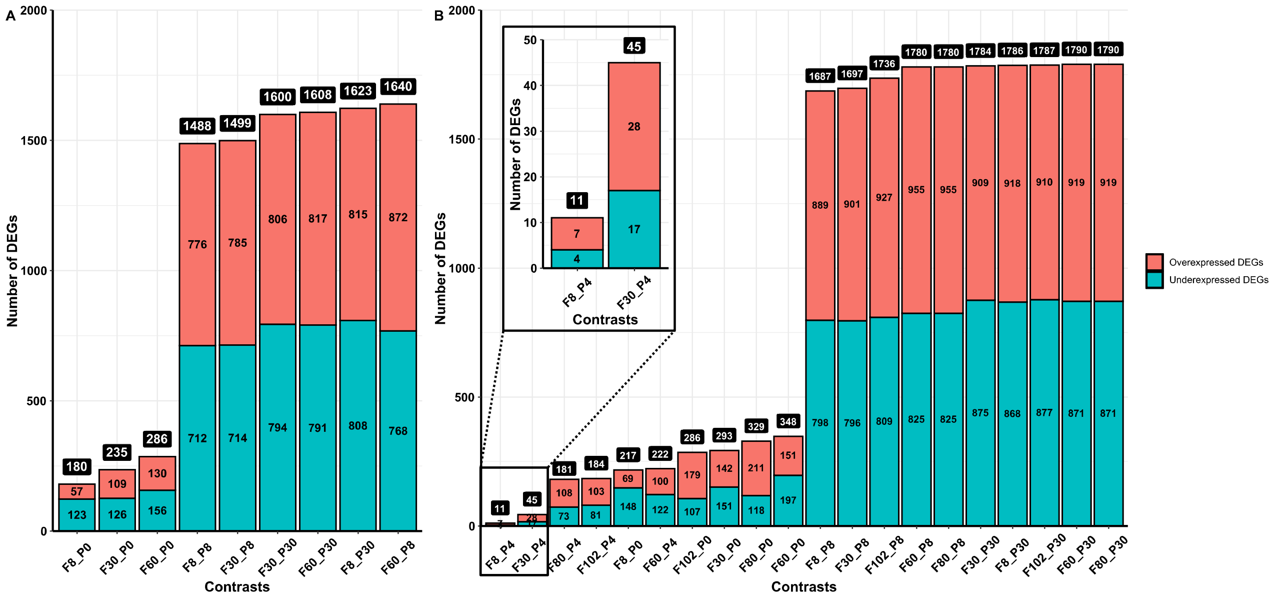

Figure S2. In (A), DNA microarray single analysis of GSE9423 dataset comparing the lager yeast CB11 strain in different times of fermentation (F) and propagation (P). In (B), DNA microarray meta-analysis comparing the lager yeast CB11 strain in different times of fermentation (F; GSE10205) and propagation (P; GSE16376). The time of point collection (in hours) is indicated

after the letters “F” and “P”. The black squares above the bars indicate the total number of DEGs observed for a given contrast. The numbers inside the blue and red bars shown the total of underexpressed and overexpressed DEGs observed in a specific contrast. The inset in the graphic (B) is a zoom of the first two bars.

Proteostasis- and chaperones-associated DEGs in DNA microarray single- and meta-analysis

The overexpressed DEGs from DNA microarray single and meta-analysis (Figure S2A and B) were filtered for proteostasis- and chaperones-associated genes using the annotated data from *Saccharomyces* Genome Database (Figure S1). The number of overexpressed proteostasis- and chaperones-associated DEGs observed in beer fermentation using DNA microarray single analysis (Figures S3A and S4A) and DNA microarray meta-analysis (Figure S5A and S6A) was similar. These DEGs were then subjected to a gene ontology (GO) analysis and the major biological processes were evaluated (Figures S3B to S6B). Data from GO analysis showed that similar biological processes were obtained after semantic reduction for both proteostasis- and chaperones-associated DEGs gathered from different DNA microarray analysis (Figures S3B to S6B).

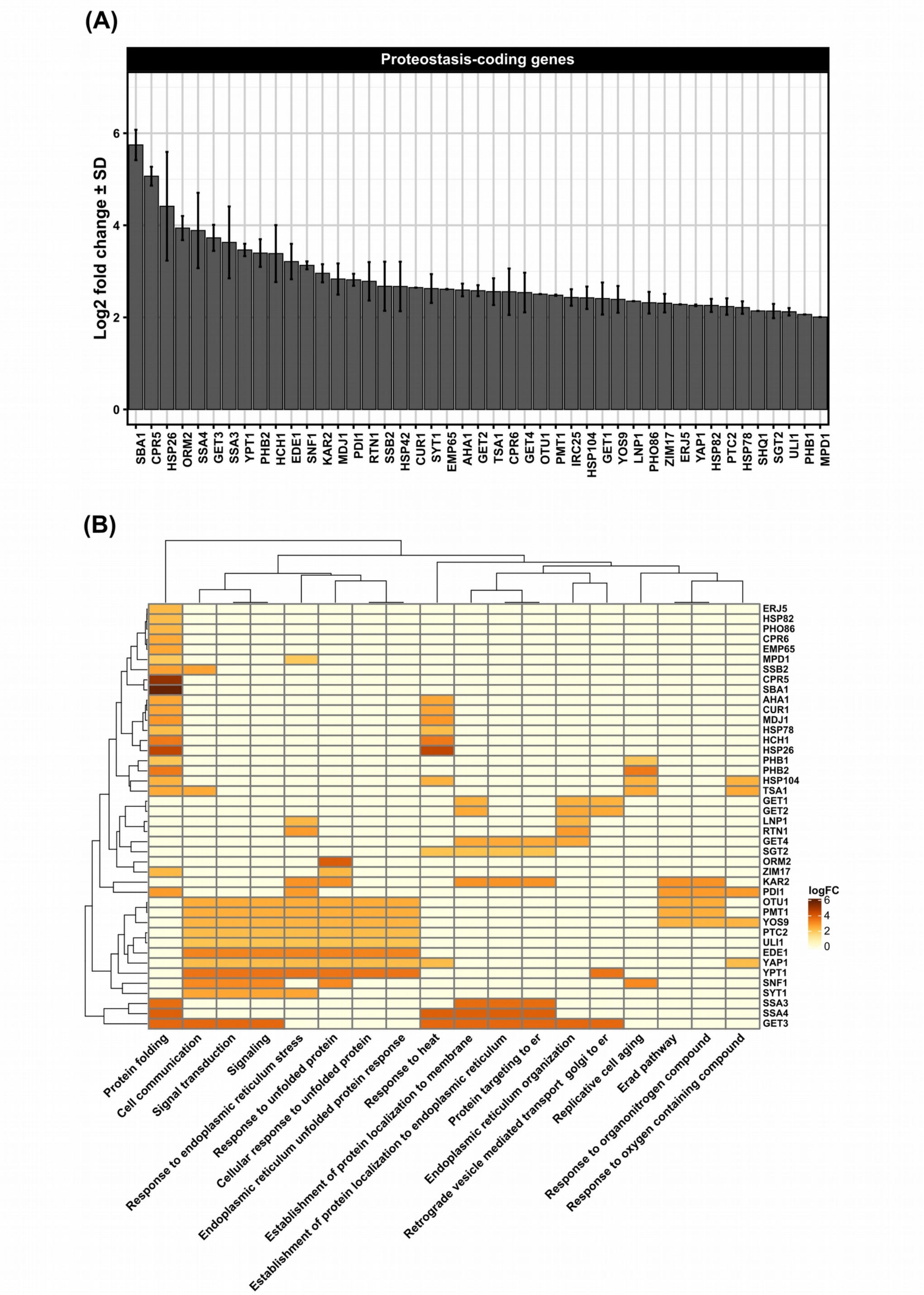

Figure S3. (A) Differentially upregulated genes from DNA microarray single analysis (GSE9423) associated with proteostasis observed in the lager yeast CB11 strain during beer fermentation, compared to the propagation step, at different times. The mean expression values are indicated by log2 fold change ± standard deviation (SD) on the y-axis and in the inset. Gene names are indicated on the x-axis. (B) Heatmap plot showing the clustered differentially upregulated genes

associated with proteostasis observed in CB11 during beer fermentation, compared to the propagation step, at different times and the associated clustered biological processes from gene ontology analysis. Heatmap rows and columns were grouped using the Euclidean distance method and complete linkage.

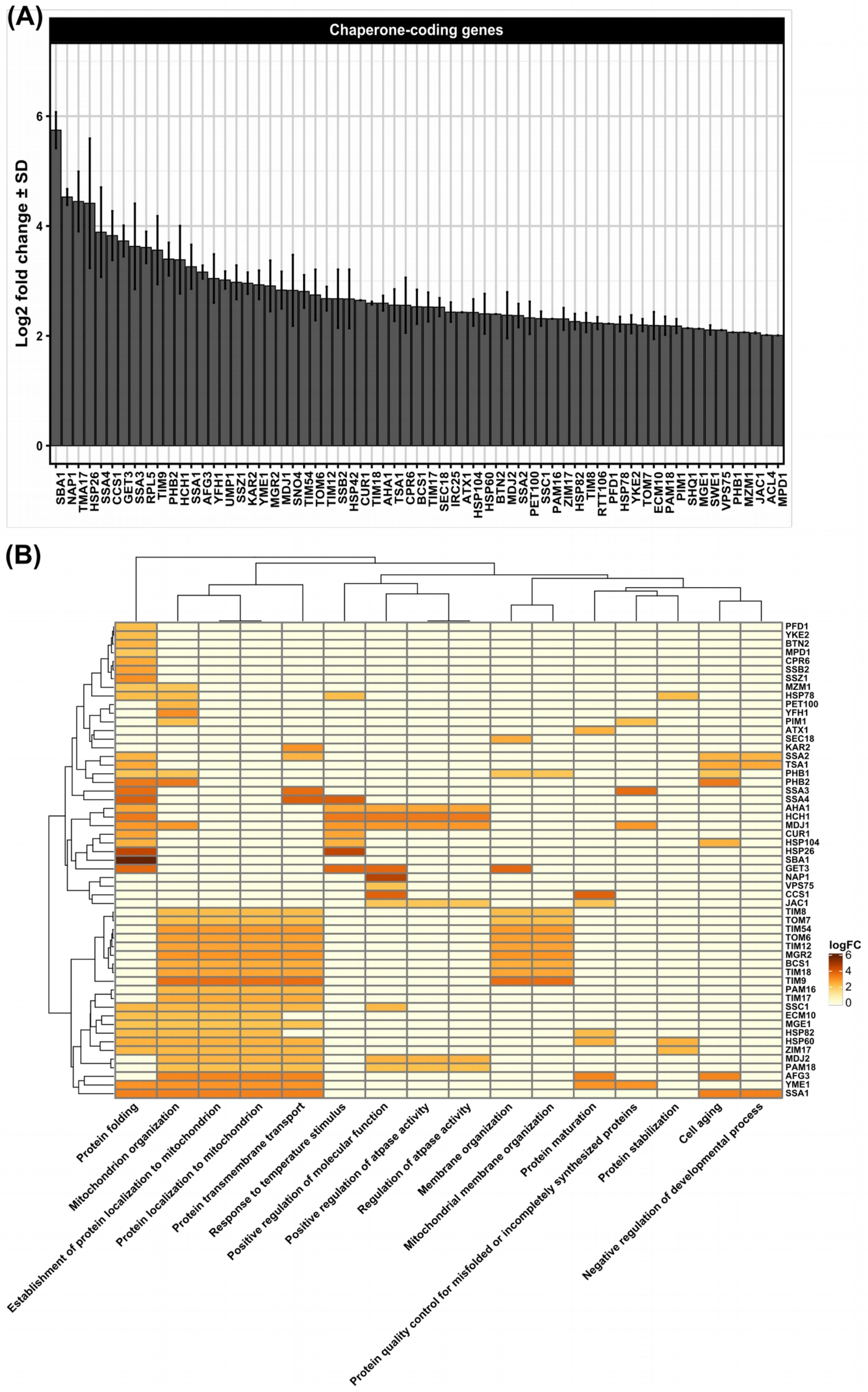

Figure S4. (A) Differentially upregulated genes from DNA microarray single analysis

(GSE9423) associated with chaperones and folding proteins observed in the lager yeast CB11 strain during beer fermentation, compared to the propagation step, at different times. The mean expression values are indicated by log2 fold change ± standard deviation (SD) on the y-axis and in the inset. Gene names are indicated on the x-axis. (B) Heatmap plot showing the clustered differentially upregulated genes associated with chaperones and folding proteins observed in CB11 during beer fermentation, compared to the propagation step, at different times and the associated clustered biological processes from gene ontology analysis. Heatmap rows and columns were grouped using the Euclidean distance method and complete linkage.

(A)

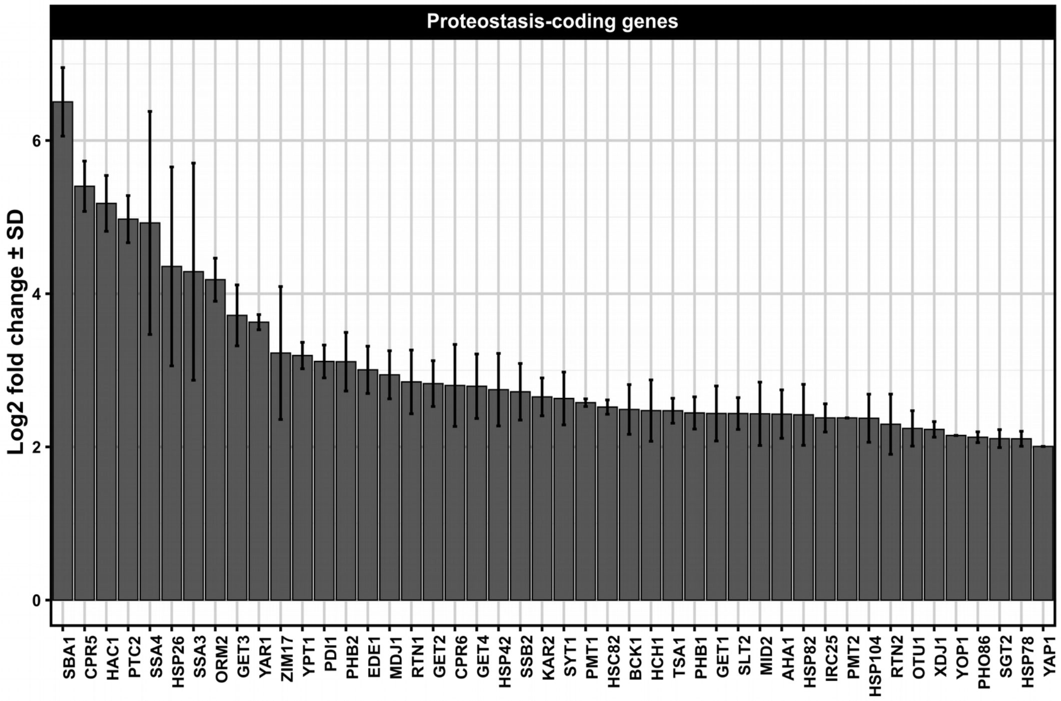

(B)

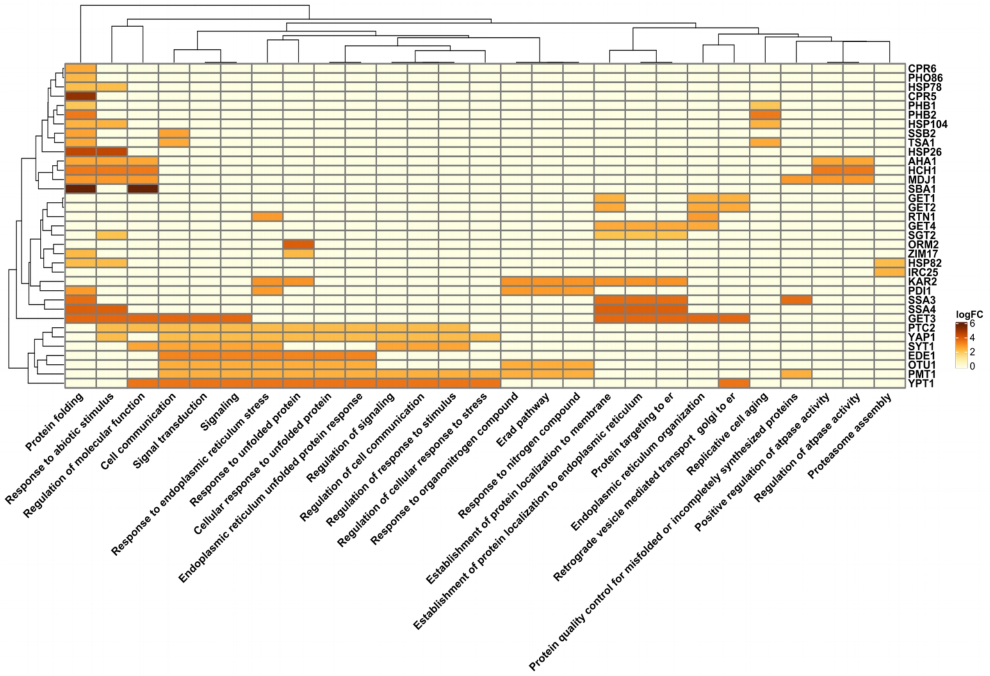

Figure S5. A) Differentially upregulated genes from DNA microarray meta-analysis (GSE10205 versus GSE16376) associated with proteostasis observed in the lager yeast CB11 strain during beer fermentation, compared to the propagation step, at different times. The mean expression

values are indicated by log2 fold change ± standard deviation (SD) on the y-axis and in the inset. Gene names are indicated on the x-axis. (B) Heatmap plot showing the clustered differentially upregulated genes associated with proteostasis observed in CB11 during beer fermentation, compared to the propagation step, at different times and the associated clustered biological processes from gene ontology analysis. Heatmap rows and columns were grouped using the Euclidean distance method and complete linkage.

(A)

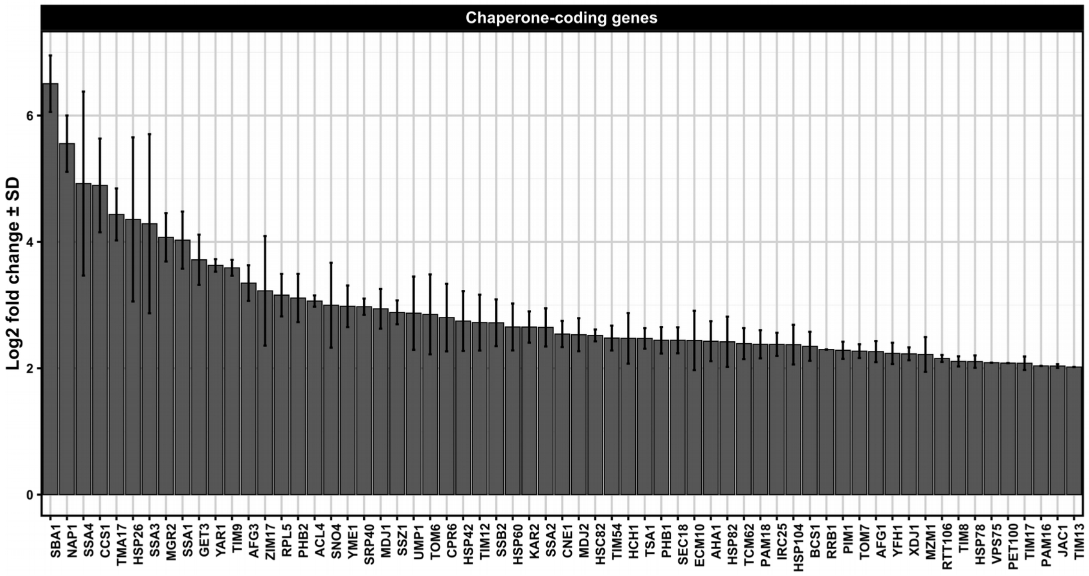

(B)

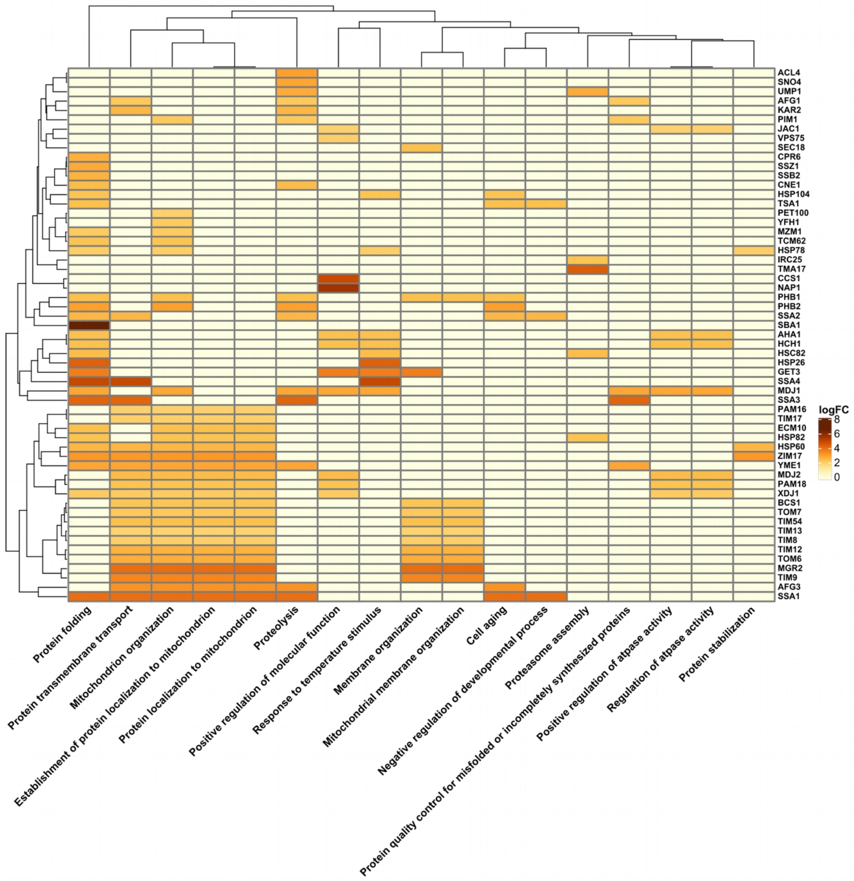

Figure S6. (A) Differentially upregulated genes from from DNA microarray meta-analysis (GSE10205 versus GSE16376) associated with chaperones and folding proteins observed in the lager yeast CB11 strain during beer fermentation, compared to the propagation step, at different

times. The mean expression values are indicated by log2 fold change ± standard deviation (SD) on the y-axis and in the inset. Gene names are indicated on the x-axis. (B) Heatmap plot showing the clustered differentially upregulated genes associated with chaperones and folding proteins observed in CB11 during beer fermentation, compared to the propagation step, at different times and the associated clustered biological processes from gene ontology analysis. Heatmap rows and columns were grouped using the Euclidean distance method and complete linkage.

Subcellular localization of proteostasis- and chaperone-associated DEGs products

The subcellular localization of proteostasis- and chaperone-associated DEGs products indicated that most of proteins can be found in cytoplasm, nucleus, ER, and mitochondria (Figures S7A to B and Figures S8A and C). In this sense, both DNA microarray analysis point to the same subcellular localization (Figures S8A and C) of different chaperone families whose members are upregulated in beer fermentation (Figures 8B and D).

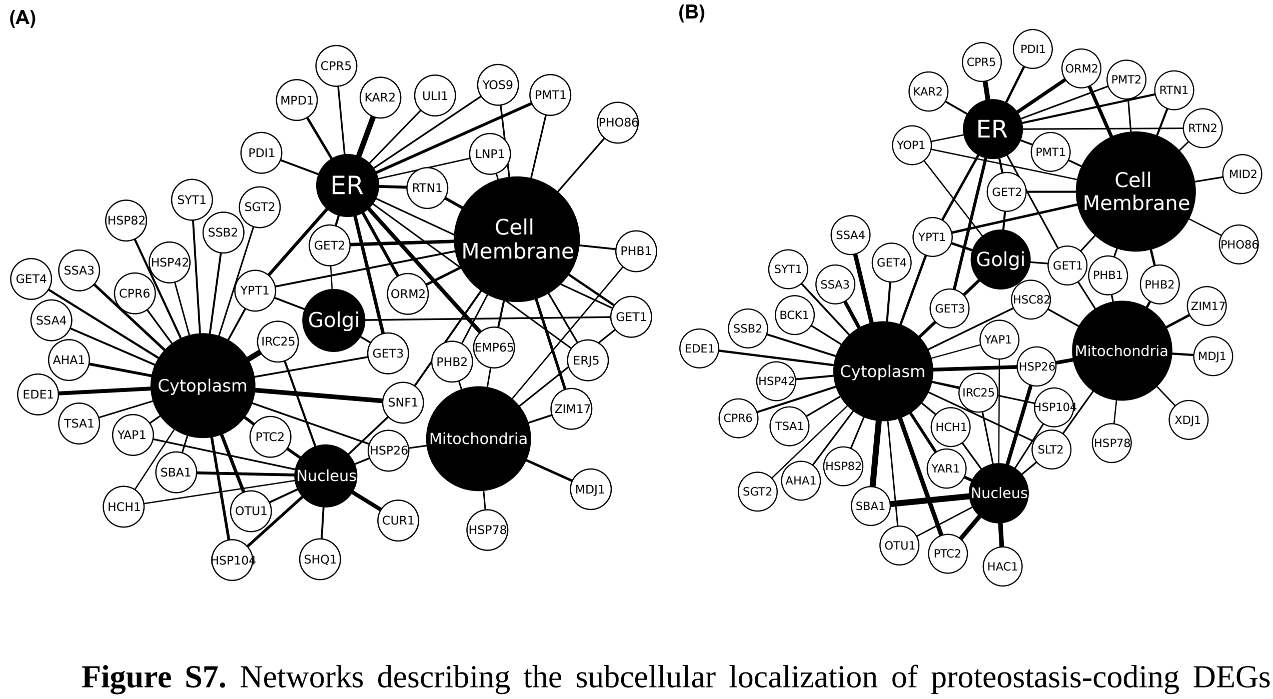

Figure S7. Networks describing the subcellular localization of proteostasis-coding DEGs obtained from DNA microarray single (GSE9423; A) and meta-analysis (GSE10205 versus GSE16376; B). The width of edges (thin to thick) is proportional to the mean logFC for each DEG

evaluated in each analysis. The diameter of nodes representing the subcellular targets do not have any biological and/or statistical significance.

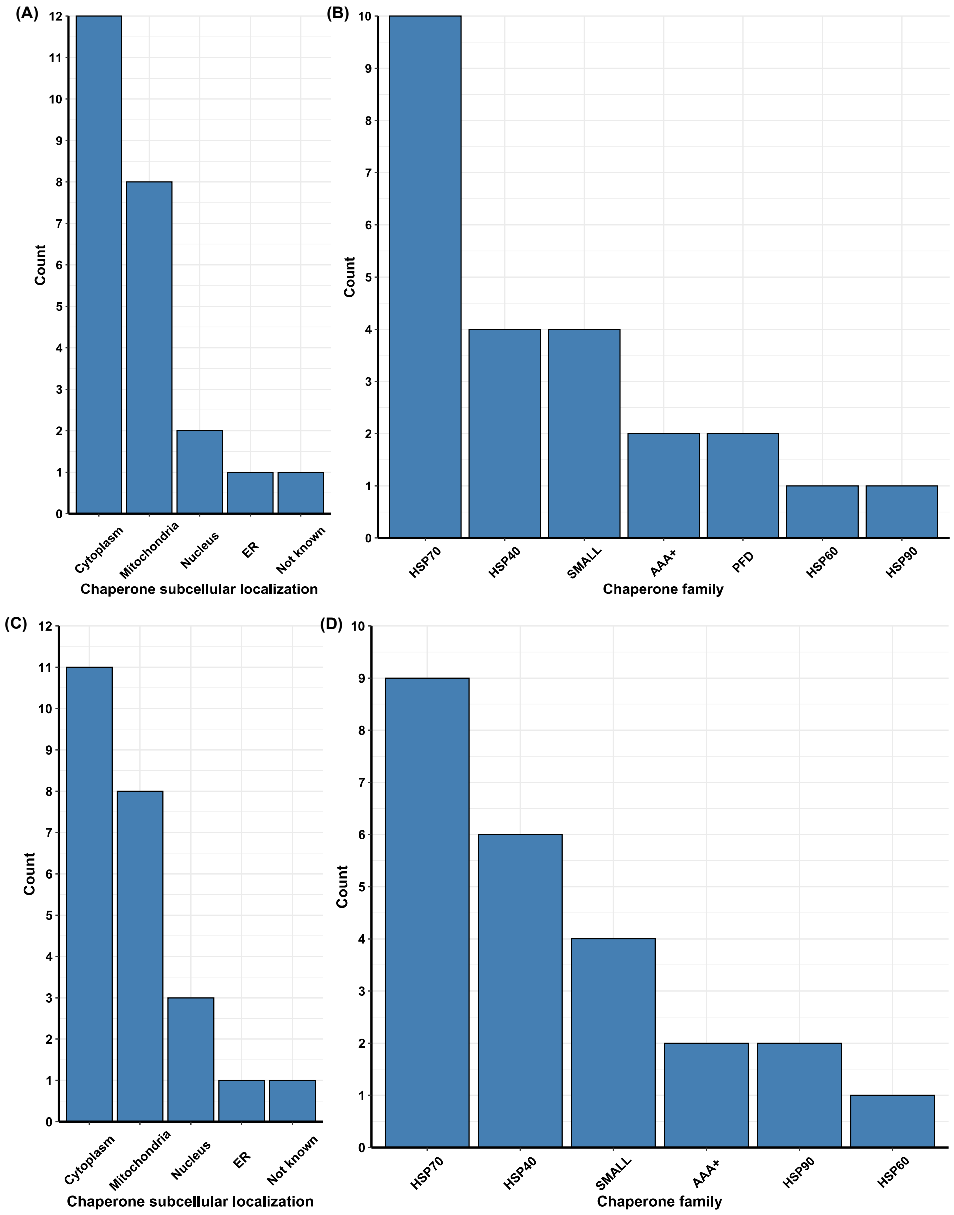

Figure S8. (A) and (C) Number of chaperones and folding protein coding genes found to be upregulated in different organelles of the lager yeast CB11 strain during beer fermentation, in comparison to yeast propagation as observed from DNA microarray single (GSE9423) and meta-

analysis (GSE10205 versus GSE16376), respectively. (B) and (D) Number of coding genes upregulated in CB11 during beer fermentation, in comparison to yeast propagation, that are linked to the major chaperone protein families as observed from DNA microarray single (GSE9423) and meta-analysis (GSE10205 versus GSE16376), respectively.

Evaluation of proteostasis- and chaperone-associated Pan-DEGs

In order to identify common upregulated proteostasis- and chaperone-associated DEGs (Pan-DEGs) in yeast lager CB11 strain during beer fermentation, DNA microarray data from single and meta-analysis were used (Figure S1). A high degree of overlap between DNA microarray analysis was observed, with 36 proteostasis-associated Pan-DEGs and 54 chaperone-associated Pan-DEGs identified (Figures S9A and B). This proteostasis- and chaperone-associated Pan-DEGs were further applied for biological processes and subcellular localization analyses (Figures 1 to 3 in the main text of the manuscript).

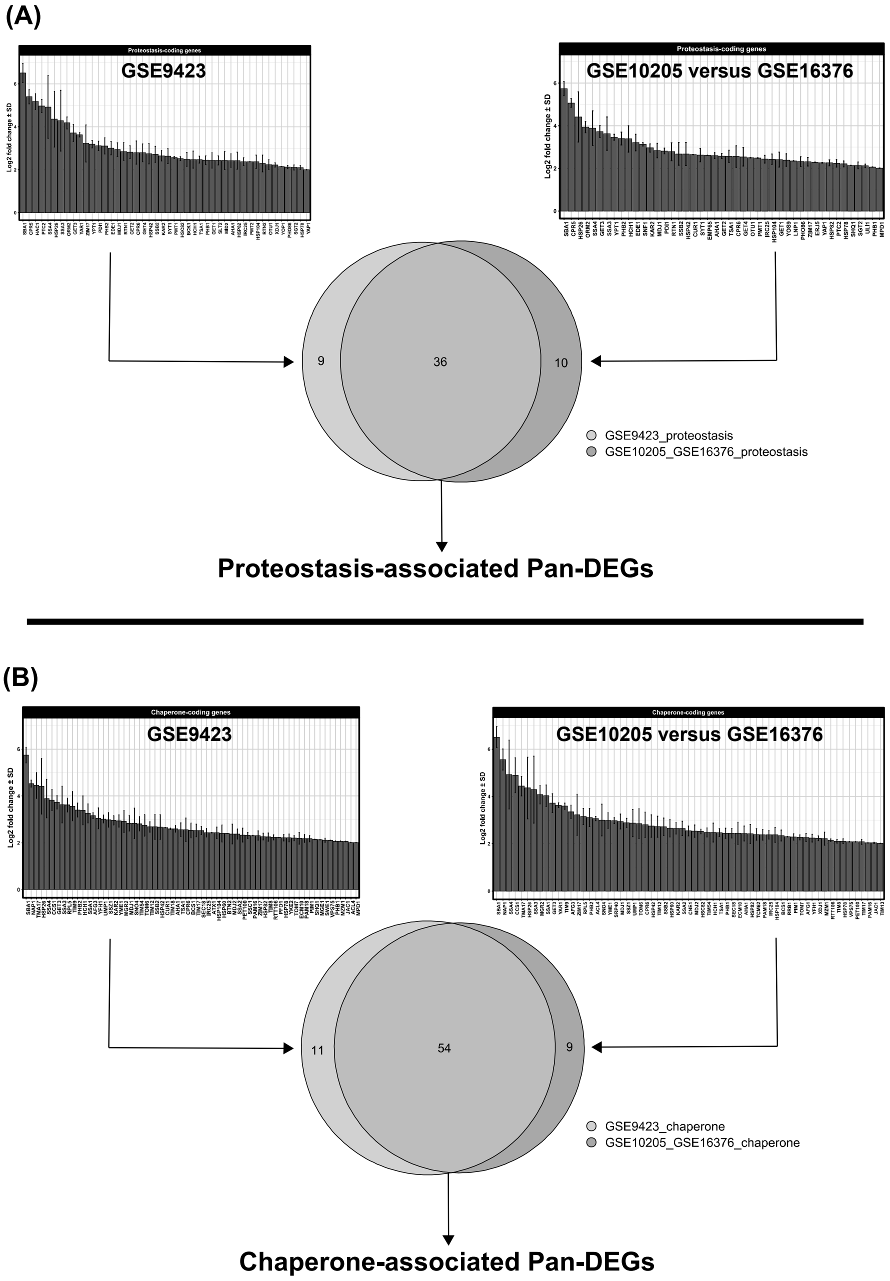

Figure S9. Evaluation of overlap degree of upregulated DEGs (Pan-DEGs) in yeast lager CB11 during beer fermentation between DNA microarray single- (GSE9423) and meta-analysis (GSE10205 versus GSE16376). (A) and (B), proteostasis- and chaperone-associated Pan-DEGs, respectively.

Additional data

Crp6p-Rpd3p-Pbp1p interaction network

A protein-protein interaction network of Crp6p with Rpd3p and Pbp1p (Figure S10) was obtained from STRING 11.0 (https://string-db.org). The following parameters were used for network prospection: *Saccharomyces cerevisiae* as selected organism; active prediction methods: databases and experiments; no more than five interactions in the first network shell; medium confidence score (0.400); evidence as meaning of network edges.

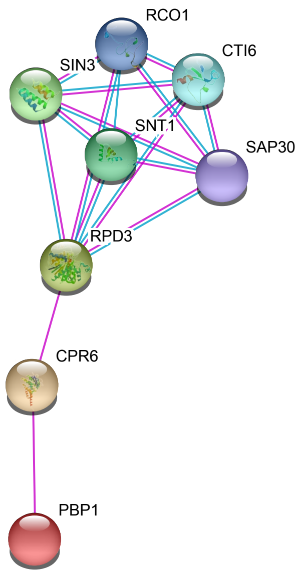

Figure S10. Protein-protein network interaction of yeast *Saccharomyces cerevisiae* Cpr6p with Rpd3p and Pbp1p. Edges color and number indicate supporting evidences from curated databases (light blue) and experiments (dark blue).

Evaluation of fatty acid-associated DEGs in Saccharomyces pastorianus CB11 strain during beer fermentation

Data from DNA single- (GSE9423) and meta-analysis (GSE10205 versus GSE16376) indicated that genes linked to fatty acid biosynthesis are upregulated in yeast lager CB11 strain during beer fernentation. In this sense, 124 upregulated fatty acid biosynthesis-associated DEGs

were observed in GSE9423 dataset (Figure S11), while 113 DEGs were overexpressed in the GSE10205 versus GSE16376 datasets (Figure S12).

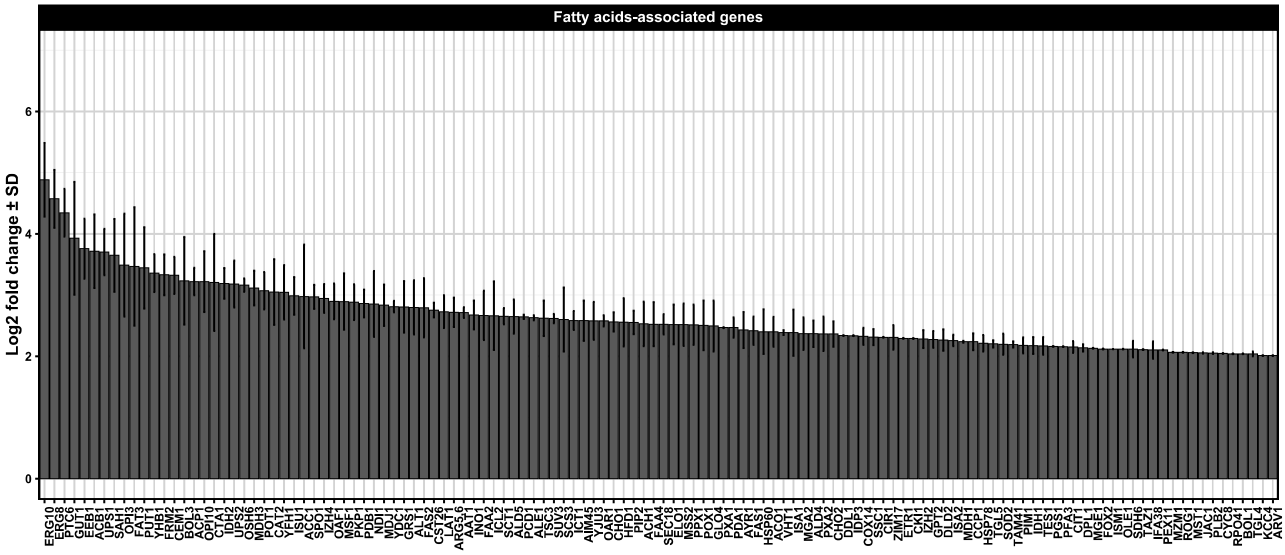

Figure S11. (A) Differentially upregulated genes from DNA microarray single analysis (GSE9423) associated with fatty acids biosynthesis observed in the lager yeast CB11 strain during beer fermentation, compared to the propagation step, at different times. The mean expression values are indicated by log2 fold change ± standard deviation (SD) on the y-axis and in the inset. Gene names are indicated on the x-axis.

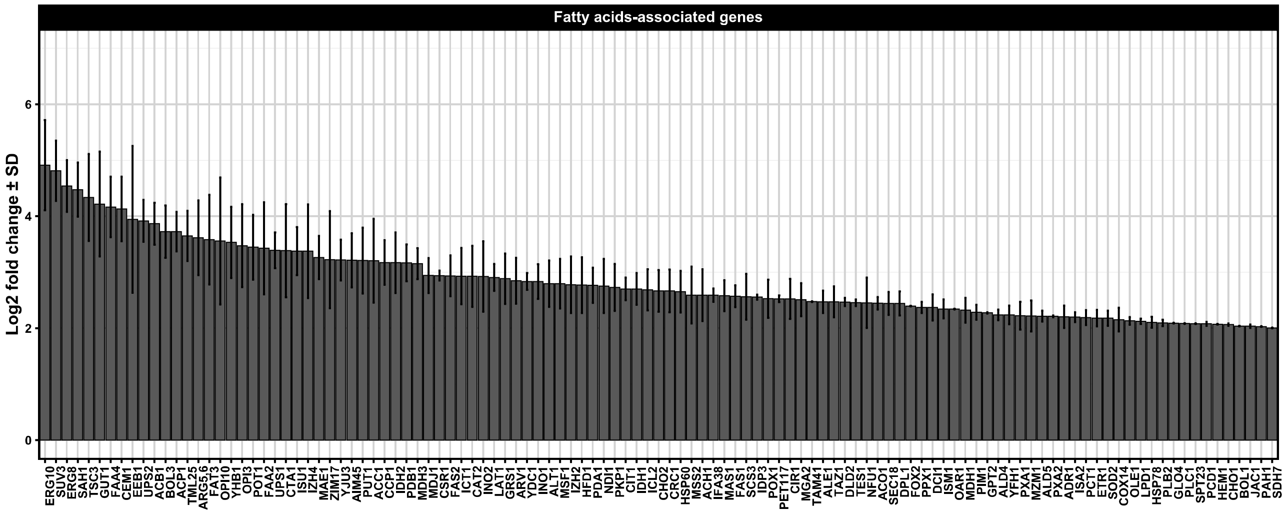

Figure S12. Differentially upregulated genes from DNA microarray meta-analysis

(GSE10205 versus GSE16376) associated with fatty acids biosynthesis observed in the lager yeast CB11 strain during beer fermentation, compared to the propagation step, at different times. The mean expression values are indicated by log2 fold change ± standard deviation (SD) on the y-axis and in the inset. Gene names are indicated on the x-axis.

References

Benjamini, Y., and Hochberg, Y. (1995). Controlling the false discovery rate: a practical and powerful approach to multiple testing. *J. R. Stat. Soc. Ser. B Methodol.* 57, 289–300.

Csardi, G., and Nepusz, T. (2006). The igraph software package for complex network research. *InterJournal* Complex Systems, 1695.

Davis, S., and Meltzer, P. S. (2007). GEOquery: a bridge between the Gene Expression Omnibus (GEO) and BioConductor. *Bioinformatics* 23, 1846–1847. doi:10.1093/bioinformatics/btm254.

Gibson, B. R., Lawrence, S. J., Boulton, C. A., Box, W. G., Graham, N. S., Linforth, R. S. T., et al. (2008). The oxidative stress response of a lager brewing yeast strain during industrial propagation and fermentation: Oxidative stress response of lager brewing yeast. *FEMS Yeast Res.* 8, 574–585. doi:10.1111/j.1567-1364.2008.00371.x.

Gu, Z., Eils, R., and Schlesner, M. (2016). Complex heatmaps reveal patterns and correlations in multidimensional genomic data. *Bioinformatics* 32, 2847–2849. doi:10.1093/bioinformatics/btw313.

Kauffmann, A., Gentleman, R., and Huber, W. (2009). arrayQualityMetrics—a bioconductor package for quality assessment of microarray data. *Bioinformatics* 25, 415–416. doi:10.1093/bioinformatics/btn647.

Ritchie, M. E., Phipson, B., Wu, D., Hu, Y., Law, C. W., Shi, W., et al. (2015). limma powers differential expression analyses for RNA-sequencing and microarray studies. *Nucleic Acids Res.* 43, e47–e47. doi:10.1093/nar/gkv007.

Shannon, P., Markiel, A., Ozier, O., Baliga, N. S., Wang, J. T., Ramage, D., et al. (2003). Cytoscape: a software environment for integrated models of biomolecular interaction networks. *Genome Res.* 13, 2498–2504. doi:10.1101/gr.1239303.

Yu, G., Li, F., Qin, Y., Bo, X., Wu, Y., and Wang, S. (2010). GOSemSim: an R package for measuring semantic similarity among GO terms and gene products. *Bioinformatics* 26, 976–978. doi:10.1093/bioinformatics/btq064.

Yu, G., Wang, L.-G., Han, Y., and He, Q.-Y. (2012). clusterProfiler: an R package for comparing biological themes among gene clusters. *OMICS J. Integr. Biol.* 16, 284–287. doi:10.1089/omi.2011.0118.
